# Supplementary material for: Effectiveness of Dry Needling of Myofascial Trigger Points in the Triceps Surae Muscles: Systematic Review
Source: Healthcare (Basel). 2022 Sep 24;10(10):1862. doi: 10.3390/healthcare10101862 (PMC9602116; doi:10.3390/healthcare10101862)
Supplement: Supplementary file 1 [file healthcare-10-01862-s001.zip › Table S1.pdf]

Table S1. Full electronic search strategy

Supplementary file 1. Complete search strategy	
**#1**	Search (“dry needling”)
**#2**	Search (“gastrocnemius” OR “soleus” OR “calf” OR “triceps surae” OR “sural triceps” OR “triceps sural”)
**#3**	Search (#1 AND #2)
